# Effect of Dentin Irrigants on Push-Out Bond Strength in Resin Cementation Protocols for Fiber Posts in Endodontically Treated Teeth: An In Vitro Study

**DOI:** 10.3390/ma17061432

**Published:** 2024-03-21

**Authors:** Sandra García-Varela, João Carlos Ramos, María José Ginzo-Villamayor, Pablo Castelo-Baz, Ramón Méndez-Díaz, Marcos Aníbal Anache-D’Abate, Tania Gancedo-Gancedo, Manuel Ruíz-Piñón, Soledad Mareque-Bueno, Benjamín José Martín-Biedma

**Affiliations:** 1 Faculty of Dentistry, University of Santiago de Compostela, Entrerríos Street, No. 1, 15705 Santiago de Compostela, Spain; mariajose.ginzo@usc.es (M.J.G.-V.); pablocastelobaz@hotmail.com (P.C.-B.); ramonclinica@hotmail.com (R.M.-D.); anache@doctor.com (M.A.A.-D.); tania.gancedo.g@gmail.com (T.G.-G.); manuelrz2@gmail.com (M.R.-P.); soledad.mareque@gmail.com (S.M.-B.); benjamin.martin@usc.es (B.J.M.-B.); 2 Faculty of Medicine, University of Coimbra, 3000-370 Coimbra, Portugal; joao.ramos@ipmd.pt

**Keywords:** bond strength, push-out, fiber post, hybrid layer, dental adhesive, dentin, chlorhexidine, EDTA, sodium hypochlorite

## Abstract

The aim of this study is to analyze the effects of different endodontic irrigants and adhesive systems on the resin bond strength of fiber post cementation. In total, 144 single-rooted, unrestored human teeth were endodontically treated and randomly divided into 12 groups according to four endodontic irrigants (distilled water as control; EDTA 17%; NaOCl 5%; chlorhexidine digluconate 2%) and three different adhesive/resin cement systems (etch-and-rinse: orthophosphoric acid, Parabond^®^ A+B/Paracore^®^; self-etch: ParaBond^®^ Non-Rinse Conditioner, Parabond^®^ A+B/Paracore^®^; Universal: Clearfil^TM^ Universal Bond/Clearfil^TM^ DC Core Plus). Forty-eight hours after post cementation, ten teeth from each group were cross-sectioned into three discs (cervical, middle and apical regions). Thirty specimens of each group (*n* = 30) were submitted to a push-out test at a crosshead speed of 1 mm/min. The remaining two teeth of each group were sectioned in the same manner, and the resin–dentin interface was evaluated using scanning electron microscopy (SEM). The results were statistically analyzed with the ANOVA test and Tukey’s test (*p* < 0,01). The adhesive protocols and post space region showed no significant effect on bond strength (*p* > 0.01). The combination of NaOCl 5% and ClearfilTM Universal Bond reduced the adhesive strength (*p* < 0.01). The NaOCl 5%, in relation to other irrigants, significantly decreased the push-out bond strength.

## 1. Introduction

Annually, millions of prefabricated fiberglass posts are sold worldwide to restore teeth that have lost a significant portion of their coronal structure to regain uniform, functional, and aesthetic tooth restoration. Intraradicular fiber posts must be cemented to prevent additional stress on the dentin [1,2,3,4].

The bond strength between the cement and dentin surface can be altered based on the relevant dentin adhesive etching strategies (etch-rinse, self-etch, and universal adhesives) [5,6,7,8].

The action of acid etching leads to collagen demineralization and hydrolytic degradation; the demineralized collagen layer may result in incomplete resin monomer infiltration [9] and may degrade due to the collagenolytic action of endogenous enzyme MMPs and cysteine cathepsins released during acid etching [10,11,12].

These effects on dentin vary depending on the type of etching system and may lead to a decrease or increase in adhesion, thereby influencing the durability of adhesive restorations. Surface treatment of dentin using various irrigating agents can induce chemical and structural alterations capable of enhancing the bond strength between dentin and dentin adhesive and cement [13,14,15,16,17,18]. The most commonly used irrigants include ethylenediaminetetraacetic acid (EDTA), chlorhexidine digluconate (CHX), and sodium hypochlorite (NaOCl). Ethylenediaminetetraacetic acid (EDTA) can demineralize dentin without altering the collagen layer and chelates both the calcium and zinc ions necessary for MMPs and hydrolases [19]. NaOCl possesses oxidative and proteolytic capabilities [20] and enhances dentin surface tension and monomer wettability [21]. Chlorhexidine digluconate (CHX) is a biguanide agent that exhibits substantivity by binding to demineralized dentin [6,22]. CHX is a nonspecific inhibitor of both MMPs and cysteine cathepsins in a dose-dependent manner [11,23] and mimics the tissue inhibitor of metalloproteinases (TIMPs) that preserves the collagen layer from degradation [24,25].

The histological and anatomical characteristics of root dentin make it susceptible to specific treatments different from other conservative dental treatments. It exhibits high density and tubule size, perpendicular orientation of tubules to the surface, a lower proportion of intertubular dentin, and a lack of intrapulpal pressure [6,10].

The aim of this study was to analyze the effects of different endodontic irrigants and adhesives systems with different acid etching strategies on the resin–dentin interface using both the push-out test and scanning electron microscopy (SEM) techniques to evaluate fiber post cementation. The objective was to establish an irrigation protocol for the root dentin space that is both safe and effective for the clinical dentist, in relation to the adhesive cementation of intraradicular posts.

Null hypotheses tested: (1) the type of irrigant, (2) the type of adhesive/cement, and (3) the region within the post space do not impact resin bond strength.

## 2. Materials and Methods

One hundred forty-four single-rooted, unrestored, and caries-free human teeth with straight root canals were extracted, due to periodontal reasons, with prior informed consent from the patient and stored in 0.5% chloramine T at 4 °C for one week. The research protocol was reviewed and approved by the Autonomous Committee of Research Ethics of Galicia (CAEIG 2015/443). The surface of the teeth was cleaned with a periodontal curette and stored in distilled water at 5 °C until use (4 weeks) [8,26].

**Figure 1:**
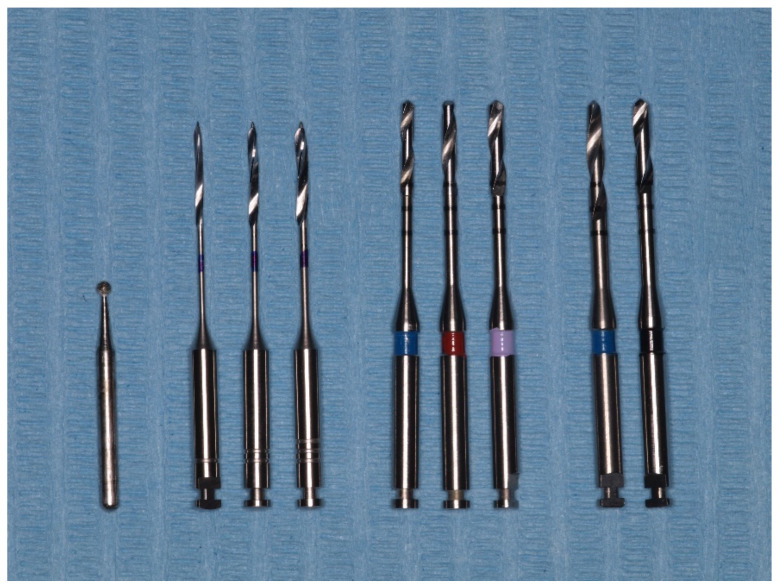
The burs and drills used to prepare the post space.

Each tooth was decoronated at the cement–enamel junction level using a diamond disc (PM 943.104.100 Ø 10 mm 0.15 mm L., Komet, Lemgo, Germany) under water cooling and treated consistently by the same operator [27]. Endodontic treatment involved the Proglider and Protaper Next System (X1, X2, X3) (Dentsply Maillefer, Ballaigues, Switzerland), 5% NaOCl (Panreac Química SLU, Barcelona, Spain), Guttacore #30, and AH Plus Jet (Dentsply DeTrey GmbH, Konstanz, Germany). The roots were provisionally sealed with Cavit G (3M Deutschland GmbH, Dental Products, Neuss, Germany). The apical foramen was sealed with dentin adhesive [26], ClearfilTM Universal Bond (Kuraray Noritake Dental Inc, Okayama, Japan), following the manufacturer’s instructions. Subsequently, the teeth were stored in distilled water at 37 °C for 24 h to allow complete cement setting (Cultura, Vivacare, diagnostic line, VIVADENT Switzerland) [26,28]. In all teeth, an 11 mm segment of the 1.5 mm diameter Tenax^®^ Fiber White (TFW) glass post (Coltène/Whaledent GmbH, Langenau, Germany) was cemented. The post section is cylindrical in its cervical and middle portions, smooth, and free from grooves or striations. The post space has a cylindrical shape, and the posts were cemented inverted using only their cylindrical portion. The post exhibited the same cross-sectional shape along the entire length of the post space, ensuring uniformity. This approach allows for a consistent cross-sectional area along the entire post space, enabling regional comparison of values and minimizing potential biases arising from taper, as investigated in the push-out test [29]. The post space was prepared with a water spray coolant at a low speed of 5000 rpm. [30]. Each drill was used to prepare 12 teeth and then discarded. The post space preparation commenced with Peeso drills 1, 2, and 3 (Maillefer Instruments Holding Sàrl, Ballaigues, Switzerland). Subsequently, cylindrical burs of 1.14 mm, 1.25 mm, and 1.4 mm (Parapost^®^ Fiber LuxTM (PFL), Coltène/Whaledent AG, Altstätten, Switzerland) were utilized, followed by use of a conical bur of 1.5 mm (TFW) and finally a cylindrical bur of 1.5 mm diameter (PFL) (Figure 1). 

The post space was rinsed with water and dried using an intraradicular aspiration system with Roeko Surgitip Endo 0.35 mm cannulas (Coltène/Whaledent AG, Altstätten, Switzerland). Finally, the adaptation of the post was verified using its cylindrical portion up to 11 mm.

The post surfaces were acid-etched using 9.6% hydrofluoric acid (Ultradent Products, Inc., South Jordan, UT, USA) for 60 s. Subsequently, they were rinsed with distilled water to remove any residual hydrofluoric acid and immersed in 96% ethyl alcohol (Laboratorios e Industrias Noriega, S.L., Oviedo, Spain) for 60 s to eliminate water remnants on the post surface. Posts were then dried using an oil-free dry air syringe. Prior to cementation, they were silanized (Ultradent Products, Inc., UT, USA) for 1 min, followed by application of oil-free dry air using a syringe [31,32].

### 2.1. Cementation and Polymerization Protocols

The teeth were randomly divided into 12 groups (Table 1). 

In all groups, 2 mL of irrigant was applied for one minute using a Canal Pro syringe with Slotted-End Tips, 27 ga. (Coltène/Whaledent AG, Altstätten, Switzerland). The irrigant was removed using a suction cannula (Roeko Surgitip-endo, Coltene Group, Altsttäten, Sweden) and standardized paper points #60 (Coltène/Whaledent GmbH, Langenau, Germany).

The following irrigants were used:

- Distilled water (Dw) (Groups: 1, 2, 3) as control.
- CanalPro™ EDTA 17% (EDTA) (Coltène/Whaledent AG, Altstätten, Switzerland) (Groups: 4, 5, 6).
- NaOCl 5% (NaOCl) (Panreac Química SLU, Barcelona, Spain) (Groups: 7, 8, 9).
- Chlorhexidine digluconate 2% in distilled water (CHX) (Groups: 10, 11, 12).

Adhesives were actively applied [33] following the manufacturer’s instructions. The cementation sequence for the groups was randomized until all groups were completed (Table 1). The adhesive techniques employed were as follows:

− Etch-rinse adhesive (Groups: 1, 4, 7, 10): 37% orthophosphoric acid (Oa) (Ivoclar Vivadent AG, Schaan, Liechtenstein) was applied in the post space for 15 s, rinsing with distilled water (15 s) using a Canal Pro syringe with Slotted-End Tips, 27 ga. (Coltène/Whaledent AG, Altstätten, Switzerland), aspiration of the water in post space with a suction tip (Roeko surgitip-endo 0.35 mm, Coltène/Whaledent AG, Altstätten, Switzerland), and drying using standardized absorbent paper points #60 (Coltène/Whaledent GmbH, Langenau, Germany). The corresponding irrigant was applied and removed as previously explained. Subsequently, the adhesive system was applied: Parabond^®^ Primer A and Primer B (PAB) (Coltène/Whaledent AG, Altstätten, Switzerland) was actively applied to the dentin surface of the post space for 30 s. A disposable mixing well (Dentsply DeTrey GmbH, Konstanz, Germany) and a microapplicator (Root Canal Applicator Tip, Dentsply DeTrey GmbH, Konstanz, Germany) were used. After the application time, excess adhesive was removed with standardized absorbent paper points #60 (Coltène/Whaledent GmbH, Langenau, Germany), and the area was air-dried using the syringe for two seconds. The combination of paper points and air was effective in removing residual water or solvent from the post space [34]. Cementation: The resin cement ParaCore^®^ (PC) (Coltène/Whaledent AG, Altstätten, Switzerland) was directly applied to the post space using its own dispensing and mixing syringe. The applicator tip was placed at the base to fill the entire receiving space of the post. As the cement was extruded, the tip was withdrawn until it exited the post space.
− Two-step self-etch adhesive (Groups: 2, 5, 8, 11): Application of the irrigant was performed as described in groups 1, 4, 7, and 10 followed by the etchant conditioning. It was performed by actively applying ParaBond^®^ Non-Rinse Conditioner (Coltène/Whaledent AG, Altstätten, Switzerland) (PNR) for 30 s using a microapplicator (Root Canal Applicator Tip, Dentsply DeTrey GmbH, Konstanz, Germany). After the application time, excess was removed with standardized absorbent paper points #60 (Coltène/Whaledent GmbH, Langenau, Germany), and the area was air-dried using the syringe for two seconds. Subsequently, the adhesive system was applied: Parabond^®^ Primer A and Primer B (PAB) (Coltène/Whaledent AG, Altstätten, Switzerland) was applied as described in groups 1, 4, 7, and 10. Cementation: ParaCore^®^ resin cement (PC) (Coltène/Whaledent AG, Altstätten, Switzerland) was applied directly to the post space as described in groups 1, 4, 7, and 10.
− Universal adhesive (Groups: 3, 6, 9, 12): Application of the corresponding irrigant was performed as described in groups 1, 4, 7, and 10 followed by the self-etch adhesive ClearfilTM Universal Bond (CUB) (Kuraray Noritake Dental Inc., Okayama, Japan). It was actively applied to the dentin surface of the post space for 10 s using a microapplicator (Root Canal Applicator Tip, Dentsply DeTrey GmbH, Konstanz, Germany). Excess adhesive and solvent were removed with standardized absorbent paper points #60 (Coltène/Whaledent GmbH, Langenau, Germany), and the area was air-dried using the syringe for five seconds. Photopolymerization of the universal dentin adhesive was performed for five seconds at 1600 mW/cm^2^ using the LED unit S.P.E.C. 3 (Coltène/Whaledent GmbH, Langenau, Germany). Cementation: The resin cement ClearfilTM DC Core Plus (CCP) (Kuraray Noritake Dental Inc., Okayama, Japan) was directly applied to the post space using its own dispensing and mixing syringe. The applicator tip was placed at the base to fill the entire receiving space of the post. As the cement was extruded, the tip was withdrawn until it exited the post space.

**Figure 2:**
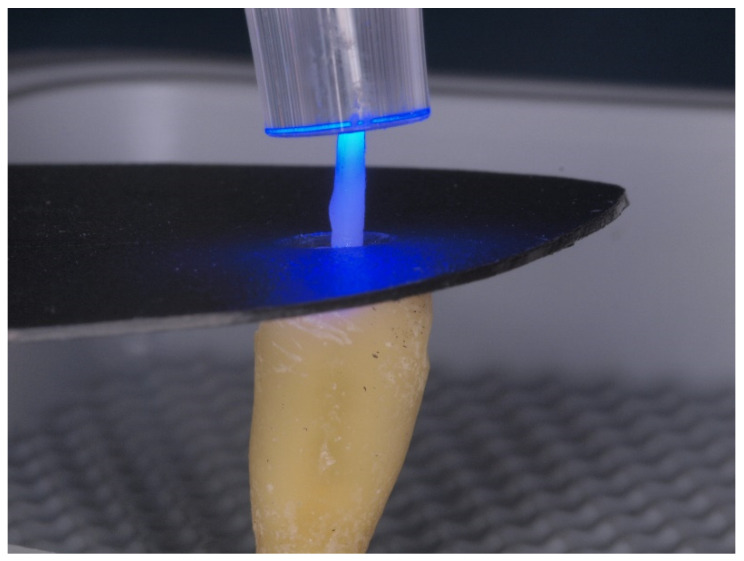
Photopolymerization of resin cement using the LED curing unit.

In all groups, once the cement was applied, the posts were positioned at the entrance of the post space, and slight digital pressure was applied until the post was fully seated in the post space (11 mm). Chemical polymerization was allowed to proceed for four minutes, followed by light activation starting from the coronal portion of the post [15]. This was undertaken using the LED curing unit S.P.E.C.3 (Coltène/Whaledent GmbH, Langenau, Germany) with an output power of 1600 mW/cm^2^ for 20 s. The curing unit’s tip was placed on the remaining part of the Tenax^®^ Fiber White post (Coltène/Whaledent AG, Altstätten, Switzerland) protruding from the root, which remained of consistent length across all teeth. To prevent light from curing the lateral root areas and to simulate oral cavity conditions, a black piece of cardboard shielded the root during photopolymerization (Figure 2). 

The cementation of the posts was undertaken following the described protocols based on manufacturer’s instructions. The variations between groups lie in the use of different irrigants and adhesive/cementation procedures. However, since the same operator performed all procedures, variations that could arise from different operators’ techniques were minimized.

Each tooth was submerged in distilled water and kept in a dark environment for 48 h at 37 °C to ensure complete cement setting [35].

### 2.2. Push-Out Test

**Figure 3:**
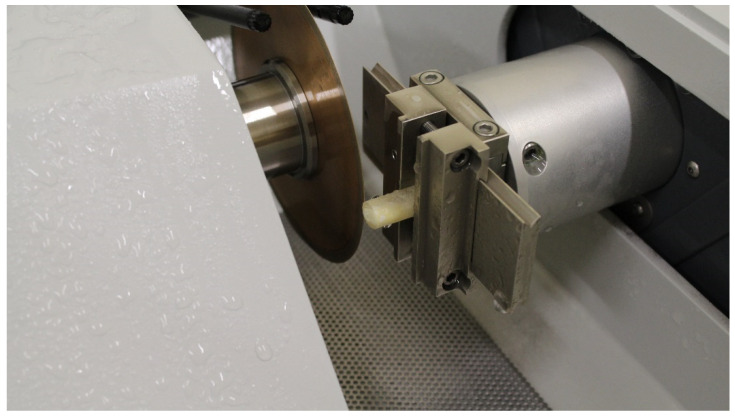
Sectioning perpendicular to the axial axis of the specimen with a precision cutting machine.

Teeth were fixed in acrylic resin cylindric blocks (10 mm diameter and 25 length) (Probase Cold Monómero, Ivoclar Vivadent AG, Schaan, Liechtenstein) keeping 0.5 mm of the cervical part of the tooth out of the resin. All teeth were vertically centered with the post parallel to the walls checked with a parallelometer (050312, Mestra, Bizkaia, Spain). Ten teeth were randomly selected from each group. Each tooth was sectioned perpendicular to its axis to ensure that the applied forces were parallel to the lateral surface of the post and the dentin, and the stress generated was comparable among the different discs obtained. A precision cutting machine (Accutom 5, Struers, Ballerup, Denmark) at 1000 rpm and with water cooling was used (Figure 3). 

Firstly, an initial cut was made to discard the coronal portion of the post at 1000 µm from the cement–enamel junction. Subsequently, three sections of 1100 µm were obtained from each tooth at distances of 1500 µm (coronal), 4500 µm (middle), and 7500 µm (apical) from the coronal portion. Discs that displayed any loss of integrity were excluded from the statistical analysis.

**Figure 4:**
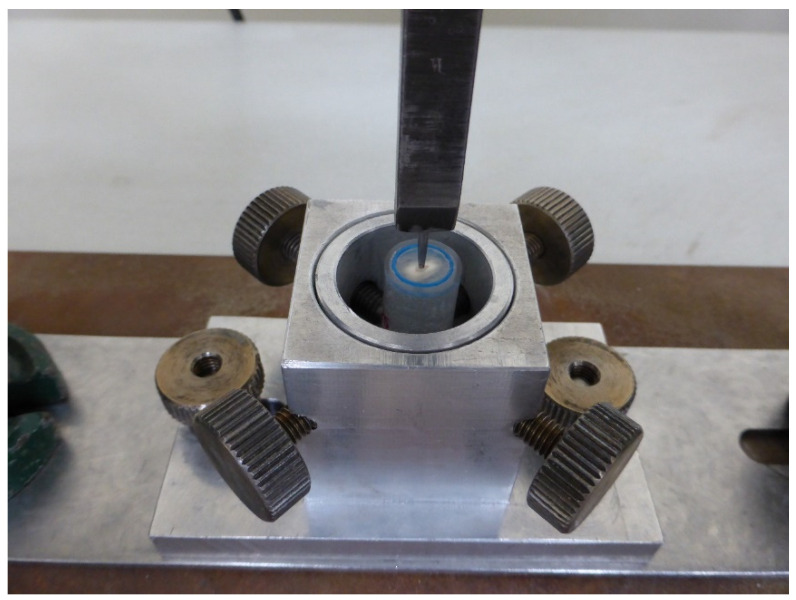
Disc placed in the universal testing machine for the push-out test. The attachment was positioned centrally on the surface post. Loads at a crosshead speed of 1 mm/min.

The discs obtained from each group (*n* = 30) were individually placed in the universal testing machine (Autograph^®^, Model AG-IS, Shimadzu Corporation, Kyoto, Japan). The attachment applying pressure onto the post had a diameter of 0.9 mm and was positioned centrally on the surface of the post (Figure 4).

The loads were perpendicular to the post’s surface and applied at a crosshead speed of 1 mm/min. The adhesive strength was calculated in MPa by dividing the maximum force required to cause specimen fracture, expressed in Newtons (N), by the adhesive surface area, expressed in square millimeters (mm^2^). The lateral bonding surface area of the cylinder was determined using the formula: Al = 2πrh, where π is a constant (3.1416), “r” is the radius of the post, and “h” is the thickness of the disc [16,36].

## 3. Results

The mean bond strength values and standard deviations are summarized in Table 2.

Regarding adhesive protocols (etch-and-rinse, two-step self-etch, and universal adhesives), no significant differences in bond strength were observed (ANOVA test, *p* > 0.01). However, significant differences were observed concerning the irrigants used (ANOVA test, *p* < 0.01). Two-by-two comparisons using the post hoc Tukey test revealed that NaOCl 5% displayed significantly lower adhesion values (*p* < 0.01).

Individual analysis of each irrigant showed no significant differences in the adhesive strength (ANOVA, *p* > 0.01). However, individual analysis of each adhesive protocol indicated that the ClearfilTM Universal Bond and ClearfilTM DC Core Plus universal systems, when combined with NaOCl 5%, significantly decreased the bond strength (Tukey’s test, *p* < 0.01).

No regional differences in bond strength were observed based on the root zones or groups values (ANOVA, *p* > 0.01) (Table 3).

### SEM Images

**Figure 5:**
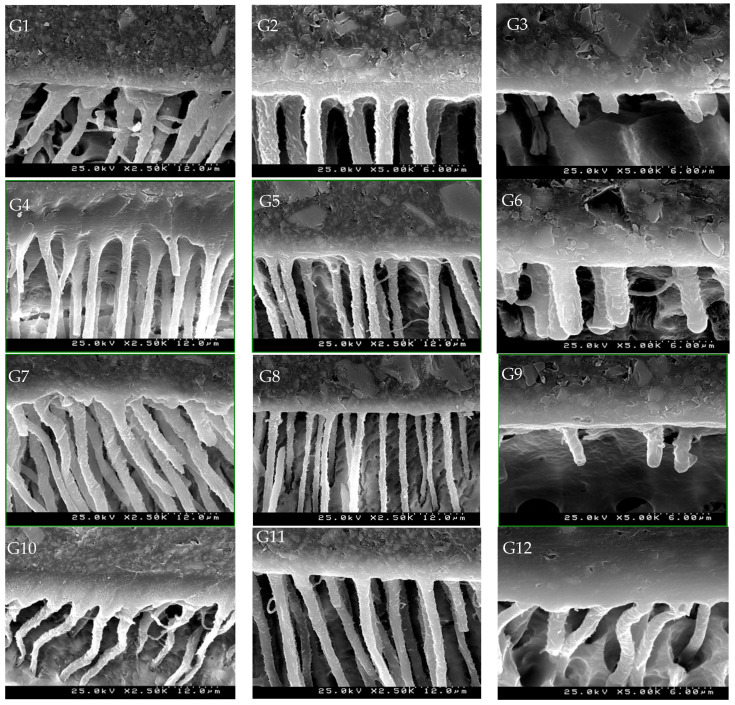
Scanning electron microscope (SEM) photomicrographs showing the cement–adhesive–dentin interface of the different groups: (**G1**) (37% orthophosphoric acid (Oa)/distilled water (Dw)/Parabond^®^ Primer A and Primer B (PAB)/resin cement ParaCore^®^ (PC)) (Oa/Dw/PAB/PC); (**G2**) (Dw/ParaBond^®^ Non-Rinse Conditioner (PNR)/PAB/PC); (**G3**) ((Dw/ClearfilTM Universal Bond (CUB)/ClearfilTM DC Core Plus (CCP)); (**G4**) (EDTA 17%/Dw/PAB/PC); (**G5**) (EDTA/PNR/PAB/PC); (**G6**) (EDTA/CUB/CCP); (**G7**) (Oa/NaOCl/PAB/PC); (**G8**) (NaOCl/PNR/PAB/PC); (**G9**) (NaOCl/CUB/CCP); (**G10**) (Oa/CHX/PAB/PC); (**G11**) (CHX/PNR/PAB/PC); (**G12**) (CHX/CUB/CCP).

The SEM images of each group are depicted in Figure 5. In the groups with an etch-and-rinse adhesive (Oa/irrigant/PAB/PC) (Figure 5G1,G4,G7,G10) or a two-step self-etch adhesive (irrigant/PNR/PAB/PC) (Figure 5G2,G5,G8,G11), numerous resin tags were observed with a thickness of 2.5 µm, featuring microporosities and lateral branches (Ø 1.2 µm) and a hybrid layer thickness between 1.5 and 4.5 µm. The hybrid layer is greater in groups treated with EDTA (Figure 5G4,G5) and CHX, especially when combining Oa/CHX 2% (Figure 5G10). However, this study indicates that there was no correlation between the increased thickness of hybrid dentin and enhanced bond strength.

Groups using etch-and-rinse adhesive show mode II peri- and intertubular demineralization. The resin tags are conical with a diameter at the tubular entry of 4–6 µm (Figure 5G1,G4,G7,G10).

In groups treated with universal adhesive (irrigant/CUB/CCP), the hybrid layer thickness is 1 µm and 1.5 µm, and the density and length of tags are lower (Figure 5G3,G6,G9). The tags are short, cylindrical, and have few lateral branches except in the group using CHX (Figure 5G12), where they are longer and exhibit greater irregularities on their surface. In the group treated with universal adhesive and NaOCl, there was a reduction in the number and length of the tags, and they do not exhibit lateral branches (Figure 5G9). Regarding the push-out test, a statistically significant decrease in adhesive strength was observed in this group (Figure 5G9). However, the bond strength achieved in other groups using ClearfilTM Universal Bond (Figure 5G,G3,G6,G12) was similar to others where NaOCl was not used, even though SEM images showed a reduced thickness of the hybrid layer and a reduced number of resin tags, which were also shorter in length.

In all groups, a basally dense zone appears, and a gradual union between dentin, adhesive, and resin cement is observed without fissures or gaps.

## 4. Discussion

Regarding in vitro studies, there has been controversy surrounding the ability of such studies to analyze the bond strength at the interface between materials and the dental surface. In vivo studies replicate the normal state of teeth, but there are serious challenges with including sufficient numbers of patients and establishing prolonged protocols to follow. Variations exist in the type of teeth used, as well as in the directions and speeds of applied forces, which would suggest that the results from various studies may not be directly comparable [37]. The experimental model we have employed allows us to assess the adhesion resistance in different samples in a standardized, repeatable, and objective manner. Such in vitro studies are crucial for evaluating and comparing different variables, as they objectively reveal the behavior of materials.

To simplify and standardize the determination of adhesive forces, the cylindrical portion of the Tenax^®^ Fiber White was cemented. The consistent cross-sectional area simplifies and standardizes the determination of adhesive forces, allowing for the comparison of adhesion values at a regional level and reducing variations due to friction [29]. Among the various mechanical tests available for assessing adherence, the push-out test is considered a viable alternative to evaluate the post–cement–dentin complex’s adherence [38]. In comparison to the tensile test, the push-out test, conducted in this study, was more reliable in measuring adhesive strength since it presents fewer premature failures, specimen preparation is simpler, and it shows acceptable data distribution variability [39,40]. The force application should be centralized on the post; otherwise, friction with parts of the dentinal walls might increase and alter the results. For this reason, the diameter of the device applying force in the push-out test was smaller than the post diameter (1.5 mm) and sufficient to avoid post deformation during load application [36]. 

To study the morphological characteristics of the resin–dentin interface in three dimensions, a scanning electron microscope (SEM) was employed. SEM allows for examination of the three-dimensional morphology of the resin–dentin interface. In this study, magnifications of 5000× were obtained. The main drawbacks of SEM lie in it being limited to observing only the surface of the sample, and the processing for its visualization may introduce artifacts [41].

An advantage for the clinical application of fiber posts would be to combine the cementation of the fiber post inside the root canal with the reconstruction of the core in a single-stage procedure. This would provide a time-saving benefit [26]. Consequently, several manufacturers offer post–core systems. This combination has been described as a secondary monoblock [42]. However, a previous study pointed out the potential negative effects of core materials in the adhesive cementation of fiber posts due to their higher filler content [43]. Post–core systems are available with different dentin conditioning protocols, such as self-etch or etch-and-rinse. Evaluation of both adhesive systems revealed contradictory results. Some studies reported no differences [44], whereas others reported higher adhesive strength in self-etch systems [45]. Other authors observed greater bond strengths in the etch-and-rinse adhesive system compared to the self-etch system [46]. The failures in homogeneity within the cement layer during the adhesive cementation of fiber posts have been described in the literature [47,48]. The potential beneficial effects of voids within the cement layer have been extensively discussed in the literature [47]. Voids can compensate for the harmful effect of a high C-factor within the post space by reducing tension in the cement. However, air bubbles can substantially weaken the composite [49]. The use of self-mixing tips can reduce the occurrence of these voids [50]. Post-endodontic restorations that are not performed immediately following the completion of endodontic treatment enable simultaneous cementation and core build-up. This may result in higher polymerization stress and a reduction in interface strength due to the higher filler percentage required for core reconstruction materials [43]. In this study, the two types of core cements (Paracore^®^ and ClearfilTM DC Core Plus) used have a similar volume and weight of inorganic filler, although it is slightly higher in ClearfilTM DC Core Plus. However, no significant differences were found in adhesive strength regarding the adhesive systems and cements used.

The tags observed in the groups where the etch-and-rinse adhesive was used exhibit increased conicity due to mode II demineralization caused by orthophosphoric acid. This acid removes the inorganic tissue inside and around the tubules, especially in the tubules perpendicular to the dentin surface [51]. The lengths of these tags range from 10 to 25 µm, except in those treated with universal adhesive, which show shorter length, lower density, and a thinner hybrid layer [52]. However, the resistance levels were similar, possibly due to the chemical binding of the MDP monomer to the residual hydroxyapatite linked to collagen fibers and the low solubility of the formed calcium salt [7,9,52,53,54,55,56,57,58,59,60,61,62].

In this study, a greater thickness of hybrid dentin does not provide increased retention [63]. This might be because a thicker layer of demineralized collagen could reduce resin infiltration, decreasing tensile strength and its elastic modulus [64,65,66,67].

SEM images revealed more pronounced microporosities and irregularities, mainly in the groups using the etch-and-rinse adhesive system (Figure 5G1,G4,G7,G10) and two-step self-etch adhesive (Figure 5G2,G5,G8,G11). These irregularities might be due to increased dentin demineralization and more irregular priming by Parabond^®^ [22]. In all groups, a basally dense area appears. Some studies suggest that it might correspond to hydroxyapatite crystals around partially demineralized collagen fibers that have been resin-infiltrated, providing greater bond strength and durability [68,69,70]. It may also correspond to hybrid dentin formation, associated with the presence of glycosaminoglycans on the surface of tags, due to their combination with the peritubular lamina limitans [68,71]. In future studies, the use of Energy Dispersive X-ray (EDX) or X-ray microtomography (XMT) may be of benefit to characterize the dentin surface. 

In this study, no significant differences were observed in the bond strength between the etch-and-rinse, two-step self-etch, or universal adhesive groups, confirming the second null hypothesis. This is likely because the evaluation took place 48 h after cementation, and adhesive surface degradation had not yet occurred.

No significant differences were observed concerning the zones of the post space (Table 3), in line with other studies [72,73,74], thus confirming the third null hypothesis. This could be attributed to complete debris removal [75,76], control of intra-canal moisture using suction tips [77], and active application of the adhesive that facilitates the diffusion of acid monomers and reduces adhesive layer degradation [33,76,78,79,80,81]. It depends on a careful execution of adhesive protocol [63]. Other factors favoring apical adhesion in this study include the use of self-mixing tips to minimize bubble formation [26] and allowing sufficient working time to prevent premature setting of adhesives and cements.

In this study, 5% NaOCl significantly affects adhesive bonding, particularly ClearfilTM Universal Bond, thus not confirming the first null hypothesis. Furthermore, 5% NaOCl alters the micromechanical interaction between adhesives and dentin by reducing calcium and phosphorus content, elastic modulus, microhardness, and flexural strength [82]. When the collagen layer is dissolved by NaOCl, the oxygen released interferes with resin polymerization [83]. The use of antioxidant/reducing agents could reverse the oxidative effects of NaOCl and facilitate adhesive monomer polymerization [84,85]. Several studies indicate that dentin type, application time, and NaOCl concentration influence self-etch adhesive bonding [16,86]. Active application of self-etch adhesives following NaOCl irrigation [33] and delayed dental restoration may enhance adhesive strength [87].

In this study, no significant differences in bond strength were observed between 17% EDTA, 2% CHX, and distilled water, consistent with the results in other studies [19,27,88,89]. An EDTA solution has relatively low surface tension, which could enhance dentin wetting and improve adhesion [90]. Its mechanism involves demineralization by carboxylic groups, removing the mineral part of dentin without denaturing the collagen layer, allowing residual apatite crystals to chemically bond with adhesive functional monomers, particularly self-etch adhesives [91]. This leads to a thinner smear layer and hybrid layer without extensive collagen denaturation, potentially increasing adhesive interface strength [27]. In this study, SEM images reveal an increased hybrid layer thickness that does not correspond to higher adhesion resistance. With prolonged exposure time, there is an increase in the width of the open dentinal tubules and the depth of the demineralization within [21]. A shorter EDTA exposure time might create a thinner hybrid layer and greater adhesion resistance. Although EDTA is not a specific chelator of calcium, it sufficiently acts as a metalloenzyme or metalloproteinase inhibitor, abolishing their catalytic activity, inactivating them, and inhibiting reactions catalyzed by these enzymes [21].

In this study, CHX does not exhibit significantly higher adhesion values compared to distilled water (Dw) or EDTA. However, it does show high absolute levels of resistance [25], particularly in the group using 37% orthophosphoric acid, consistent with other studies [23,92,93,94,95], while also displaying a thicker hybrid layer. This might be attributed to CHX’s significant substantivity, enabling it to bind electrostatically and reversibly to mineralized and, notably, demineralized dentin [22]. CHX increases dentin surface energy [92,96,97], thereby enhancing resin tubular infiltration [98,99] and reducing collagen degradation within the first 24 h [100,101]. In comparison to NaOCl, it achieves higher adhesive forces, possibly due to its non-oxidizing nature [82,102]. Its action is dose-dependent [11] and diminishes in the presence of calcium chloride, suggesting that its chelating properties may be crucial in improving adhesion by inactivating MMPs [95,103].

## 5. Study Limitations

− Limited scope regarding newer materials: this study focuses on specific irrigants and adhesive systems.
− Short-term analysis: this study assesses bond strength shortly after cementation without considering long-term durability.
− Lack of diversity in tooth selection: using only single-rooted, unrestored human teeth may limit the applicability of the results to a broader range of clinical scenarios.
− Potential for bias in sample preparation: the methodology relies on manual processes, such as the application of adhesives and the preparation of teeth, which could introduce variability affecting this study’s outcomes.

## 6. Conclusions

Within the limitations of the present study, the following conclusions were made:

− 5% NaOCl for one minute significantly reduces adhesive strength compared to the other irrigants.
− 5% NaOCl for one minute significantly reduces the adhesive strength of Clearfil^TM^ Universal Bond.
− There were no significant differences in adhesion between the three adhesive procedures studied.
− There were no significant differences in adhesion between the irrigants: distilled water, EDTA 17%, and chlorhexidine digluconate 2% groups.
− There were no significant differences in adhesion among the coronal, middle, and apical regions of the post space.

According to the results of this study, the use of NaOCl as an irrigant prior to the adhesive cementation of intraradicular posts is not recommended. However, further studies should be conducted to prove the results clinically.

## Figures and Tables

**Table 1:** Different groups under study according to irrigation and cementation protocols with 12 teeth in each group (*n* = 12).

	Orthophosphoric Acid 37% PARABOND A+B PARACORE	Non-Rinse Conditioner PARABOND A+B PARACORE	CLEARFIL UNIVERSAL CLEARFIL DC CORE PLUS
Distilled water (control)	G1	G2	G3
EDTA 17%	G4	G5	G6
NaOCl5%	G7	G8	G9
CHX 2%	G10	G11	G12

**Table 2:** Mean results with standard deviation for the push-out test values in MPa. Mean values with the same letters are not significantly different at *p* < 0.01. Superscripts compare from left to right (rows) and subscripts compare from top to bottom (columns). Every Group of Study (*n* = 30 discs).

	Etch-and-Rinse Adhesive Orthophosphoric Acid 37% Parabond A+B Paracore	Two Steps Self-Etch Adhesive ParaBond^®^ Non-Rinse Conditioner Parabond A+B Paracore	Universal Adhesive Clearfil Universal Clearfil DC Core Plus	Total
Distilled Water (control)	G1 = 16,760 ^a^_a_ ± 4.43	G2 = 16,261 ^a^_a_ ± 5.88	G3 = 17,067 ^a^_bc_ ± 4.33	16,696 _ac_
EDTA 17%	G4 = 17,069 ^a^_a_ ± 5.03	G5 = 14,951 ^a^_a_ ± 5.14	G6 = 16,663 ^a^_ac_ ± 4.84	16,228 _ab_
NaOCl 5%	G7 = 14,606 ^a^_a_ ± 3.81	G8 = 14,095 ^a^_a_ ± 4.64	G9 = 12,919 ^a^ ± 3.79	13,873
CHX 2%	G10 = 17,304 ^a^_a_ ± 5.95	G11= 16,479 ^a^_a_ ± 3.04	G12 = 16,604 ^a^_ab_ ± 4.58	16,796 _bc_
Total	16,435 ^a^	15,447 ^a^	15,813 ^a^	15,898 ^a^

**Table 3:** Mean resistance (MPa) in the push-out test for the coronal, middle, and apical regions of the post space.

	Coronal	Media	Apical
(MPa)	16,198	16,333	15,163

## Data Availability

The original contributions presented in the study are included in the article, further inquiries can be directed to the corresponding author.
